# Supplementary material for: Pyruvate Dehydrogenase Kinase 1 inhibition mediated oxidative phosphorylation enhancement in cartilage promotes osteoarthritis progression
Source: BMC Musculoskelet Disord. 2023 Jul 20;24:597. doi: 10.1186/s12891-023-06585-6 (PMC10357736; doi:10.1186/s12891-023-06585-6)
Supplement: Supplementary file 1 — Additional file 1. [file 12891_2023_6585_MOESM1_ESM.zip › BP.Gsea.1653623667859/GOBP_POSITIVE_REGULATION_OF_TRANSCRIPTION_BY_RNA_POLYMERASE_I.html]

Details for gene set GOBP\_POSITIVE\_REGULATION\_OF\_TRANSCRIPTION\_BY\_RNA\_POLYMERASE\_I[GSEA]

|  || Dataset | 55457\_collapsed\_to\_symbols.cls.cls #OA\_versus\_normal.cls.cls #OA\_versus\_normal\_repos |
| Phenotype | cls.cls#OA\_versus\_normal\_repos |
| Upregulated in class | normal |
| GeneSet | GOBP\_POSITIVE\_REGULATION\_OF\_TRANSCRIPTION\_BY\_RNA\_POLYMERASE\_I |
| Enrichment Score (ES) | -0.5752893 |
| Normalized Enrichment Score (NES) | -1.7054274 |
| Nominal p-value | 0.01208981 |
| FDR q-value | 0.12799658 |
| FWER p-Value | 1.0 |
Table: GSEA Results Summary

  

Fig 1: Enrichment plot: GOBP\_POSITIVE\_REGULATION\_OF\_TRANSCRIPTION\_BY\_RNA\_POLYMERASE\_I      
 Profile of the Running ES Score & Positions of GeneSet Members on the Rank Ordered List

  

| SYMBOL | TITLE | RANK IN GENE LIST | RANK METRIC SCORE | RUNNING ES | CORE ENRICHMENT || 1 | ERBB2 | erb-b2 receptor tyrosine kinase 2 [Source:HGNC Symbol;Acc:HGNC:3430] | 963 | 0.526 | -0.0010 | No |
| 2 | PIH1D1 | PIH1 domain containing 1 [Source:HGNC Symbol;Acc:HGNC:26075] | 2667 | 0.283 | -0.0935 | No |
| 3 | NOL11 | nucleolar protein 11 [Source:HGNC Symbol;Acc:HGNC:24557] | 5984 | -0.003 | -0.3505 | No |
| 4 | BNC1 | basonuclin 1 [Source:HGNC Symbol;Acc:HGNC:1081] | 6023 | -0.006 | -0.3525 | No |
| 5 | MARS1 | methionyl-tRNA synthetase 1 [Source:HGNC Symbol;Acc:HGNC:6898] | 6400 | -0.035 | -0.3768 | No |
| 6 | PWP1 | "PWP1 homolog, endonuclein [Source:HGNC Symbol;Acc:HGNC:17015]" | 8724 | -0.211 | -0.5276 | No |
| 7 | HEATR1 | HEAT repeat containing 1 [Source:HGNC Symbol;Acc:HGNC:25517] | 9339 | -0.259 | -0.5391 | Yes |
| 8 | IPPK | inositol-pentakisphosphate 2-kinase [Source:HGNC Symbol;Acc:HGNC:14645] | 9547 | -0.278 | -0.5162 | Yes |
| 9 | SMARCA4 | "SWI/SNF related, matrix associated, actin dependent regulator of chromatin, subfamily a, member 4 [Source:HGNC Symbol;Acc:HGNC:11100]" | 10009 | -0.322 | -0.5068 | Yes |
| 10 | EIF2AK3 | eukaryotic translation initiation factor 2 alpha kinase 3 [Source:HGNC Symbol;Acc:HGNC:3255] | 10025 | -0.324 | -0.4626 | Yes |
| 11 | SMARCB1 | "SWI/SNF related, matrix associated, actin dependent regulator of chromatin, subfamily b, member 1 [Source:HGNC Symbol;Acc:HGNC:11103]" | 10117 | -0.333 | -0.4230 | Yes |
| 12 | PHF8 | PHD finger protein 8 [Source:HGNC Symbol;Acc:HGNC:20672] | 11592 | -0.514 | -0.4655 | Yes |
| 13 | ATF4 | activating transcription factor 4 [Source:HGNC Symbol;Acc:HGNC:786] | 11999 | -0.593 | -0.4139 | Yes |
| 14 | MTOR | mechanistic target of rapamycin kinase [Source:HGNC Symbol;Acc:HGNC:3942] | 12047 | -0.605 | -0.3328 | Yes |
| 15 | NCL | nucleolin [Source:HGNC Symbol;Acc:HGNC:7667] | 12255 | -0.662 | -0.2561 | Yes |
| 16 | UBTF | upstream binding transcription factor [Source:HGNC Symbol;Acc:HGNC:12511] | 12261 | -0.665 | -0.1633 | Yes |
| 17 | WDR43 | WD repeat domain 43 [Source:HGNC Symbol;Acc:HGNC:28945] | 12424 | -0.716 | -0.0756 | Yes |
| 18 | DDX11 | DEAD/H-box helicase 11 [Source:HGNC Symbol;Acc:HGNC:2736] | 12607 | -0.803 | 0.0227 | Yes |
Table: GSEA details [plain text format]

  

Fig 2: GOBP\_POSITIVE\_REGULATION\_OF\_TRANSCRIPTION\_BY\_RNA\_POLYMERASE\_I      
 Blue-Pink O' Gram in the Space of the Analyzed GeneSet

  

Fig 3: GOBP\_POSITIVE\_REGULATION\_OF\_TRANSCRIPTION\_BY\_RNA\_POLYMERASE\_I: Random ES distribution      
 Gene set null distribution of ES for **GOBP\_POSITIVE\_REGULATION\_OF\_TRANSCRIPTION\_BY\_RNA\_POLYMERASE\_I**
